# Prognostic Value of *RNASEH2A*-, *CDK1*-, and *CD151*-Related Pathway Gene Profiling for Kidney Cancers

**DOI:** 10.3390/ijms19061586

**Published:** 2018-05-28

**Authors:** Chin-An Yang, Hsi-Yuan Huang, Ju-Chen Yen, Jan-Gowth Chang

**Affiliations:** 1 Department of Laboratory Medicine, China Medical University Hospital, Taichung, Taiwan; #2 Yude Road, Taichung 40447, Taiwan; yangginan81@gmail.com (C.-A.Y.); t29206@mail.cmuh.org.tw (H.-Y.H.); 2 Division of General Pediatrics, China Medical University Children’s Hospital, Taichung 40447, Taiwan; 3 College of Medicine, China Medical University, Taichung 40402, Taiwan; 4 Epigenome Research Center, China Medical University Hospital, Taichung 40447, Taiwan; t24399@mail.cmuh.org.tw

**Keywords:** *RNASEH2A*, *CDK1*, *CD151*, synthetic dosage lethality, renal cell carcinoma

## Abstract

The nucleotide degrading enzyme gene *RNASEH2A* (ribonuclease H2 subunit A) has been found to be overexpressed in cancers. Our aim was to understand the role of *RNASEH2A* in cancer prognostication and to establish a scoring system based on the expressions of genes interacting with *RNASEH2A.* We screened the nucleotide degrading enzyme gene expression in RNAseq data of 14 cancer types derived from The Cancer Genome Atlas (TCGA) and found that *RNASEH2A* overexpression was associated with poor patient survival only in renal cell carcinomas (RCCs). Further cluster analyses of samples with poor outcomes revealed that cluster of differentiation 151 (*CD151*) upregulation correlated with low cyclin dependent kinase 1 (*CDK1*) and high *RNASEH2A* expression. The combination of low *CD151* expression and high *RNASEH2A* expression resulted in impaired proliferation in four kidney cancer cell lines, suggesting potential synthetic dosage lethality (SDL) interactions between the two genes. A prognostication scoring system was established based on the expression levels of *RNASEH2A-*, *CDK1-*, and *CD151-*related genes, which could effectively predict the overall survival in a TCGA clear cell RCC cohort (*n* = 533, 995.3 versus 2242.2 days, *p* < 0.0001), in another clear cell renal cell carcinoma (ccRCC) cohort E-GEOD-22541 (*n* = 44, 390.0 versus 1889.2 days, *p* = 0.0007), and in a TCGA papillary RCC (pRCC) cohort (*n* = 287, 741.6 versus 1623.7 days, *p* < 0.0001). Our results provide a clinically applicable prognostication scoring system for renal cancers.

## 1. Background

Nucleotide metabolism homeostasis is important for the balance of cell proliferation, DNA replication, and genome stability [1]. Genomic instability facilitates tumor initiation and progression. However, the roles of nucleotide degrading enzymes in tumorigenesis are not fully understood. Loss-of-function mutations in the nucleotide degrading enzymes sterile alpha motif (SAM) domain and HD domain-containing protein 1 (*SAMHD1*), Three prime repair exonuclease 1 (*TREX1*), and ribonuclease H2 subunits A, B, and C (*RNASEH2A*, *RNASEH2B*, *RNASEH2C*) in human germline mainly result in a hyper-inflammatory Aicardi–Goutières syndrome (AGS), and in the development of malignancy in AGS patient is rare [2,3,4]. Our previous study on a Taiwanese colon cancer cohort showed that, in contrast to the downregulation of *TREX1*, *SAMHD1*, and *RNASEH2C*, *RNASEH2A* expression was found to be higher in tumor tissues when compared to paired normal parts [5]. Interestingly, *RNASEH2A* has also been reported to be upregulated in a variety of cancers, including breast, bladder, brain, prostate, head and neck cancers, seminomas, and leukemia [6].

*RNASEH2A* is the main catalytic unit of ribonuclease H2 (RNase H2), which degrades the ribonucleotide that is mis-incorporated into the DNA–DNA complex and cleaves the lagging-strand Okazaki fragment RNA primers from the DNA:RNA duplex during DNA replication [7]. Recently, *RNASEH2A* was found to have functions other than that of nucleotide degrading enzyme. For example, it was found that it could promote proliferation in sarcoma, breast cancer, and glioma cell lines, suggesting that it plays a role in cancer progression [6]. In prostate cancer, a positive correlation between *RNASEH2A* expression and cancer aggressiveness has been reported [8]. However, we did not detect an association between high *RNASEH2A* levels and colon cancer prognosis in a previous Taiwanese cohort [5]. The prognostic value of *RNASEH2A* on patient survival in other cancer types also remains unclear. Furthermore, regulatory pathways that interact with *RNASEH2A* to determine the growth of tumor cells may exist. It has been reported that certain genes have synthetic dosage lethality (SDL) interactions with genes that are frequently overexpressed in tumors and that inhibition of the SDL partners can decrease cancer proliferation [9]. In the present study, we comprehensively analyzed pan-cancer gene expression profiles that were correlated with *RNASEH2A* upregulation and we combined stratified survival analyses to identify potential regulatory genes which, together with *RNASEH2A*, provide great prognostic value for predicting the overall survival rate of cancer patients.

## 2. Results

### 2.1. Pan-Cancer Analysis of Nucleotide Degrading Enzyme Genes

**Figure 1:**
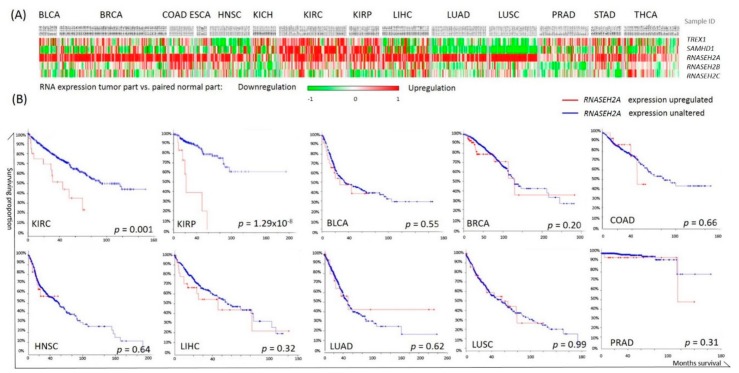
Cancer nucleotide degrading enzyme gene expressions and their association with patient overall survival. (**A**) *TREX1*, *SAMHD1*, *RNASEH2A*, *RNASEH2B*, and *RNASEH2C* expression in each subject as compared with the corresponding normal tissues. Red indicates higher expression in tumor tissue and green indicates lower expression in cancer, as compared to normal tissues. Each column represents a patient sample of The Cancer Genome Atlas (TCGA) cancer cohorts. (**B**) Kaplan-Meier analyses of overall survival in patients with higher *RNASEH2A* expression (red line) or with unaltered *RNASEH2A* expression (blue line) calculated by cBioPortal software; *p* values were calculated by log-rank tests.

We first screened the messenger RNA (mRNA) expression of the nucleotide degrading enzyme genes *TREX1*, *SAMHD1*, *RNASEH2A*, *RNASEH2B*, and *RNASEH2C* in 14 cancer types using TCGA RNAseq data. Upregulation of *RNASEH2A* in cancerous tissues compared with normal tissues was observed across cancer types, except for chromophobe kidney cancer (KICH) (Figure 1A). However, Kaplan–Meier survival analyses of these cancers using the cBioPortal software showed that higher *RNASEH2A* expression was associated with a poor prognosis only in renal carcinomas, Figure 1B.

### 2.2. RNASEH2A Network Analysis and Identification of an Alternative Pathway Promoting Tumor Growth in CDK1-Low Tumors

**Figure 2:**
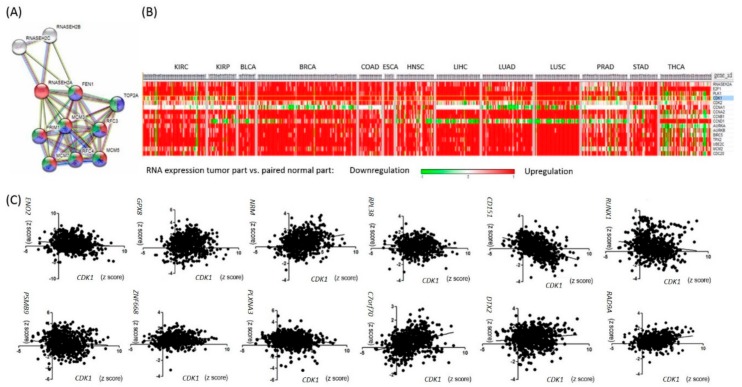
Genes correlated with *RNASEH2A* expression. (**A**) *RANSEH2A* network analysis via STRING. Genes are enriched in pathways of DNA replication (red), mitotic cell cycle (blue), and cell cycle progression (green). (**B**) Expression profile of cell cycle-related genes in TCGA 13 cancer cohorts. *CDK1* is marked in blue. (**C**) Pan-cancer gene expression analyses plotted in correlation with *CDK1* expression.

In order to investigate the additional genes contributing to survival in *RNASEH2A-*overexpressing tumors, we performed *RNASEH2A* network analysis using STRING version 10.5 1 [10]. In addition to DNA replication, *RNASEH2A* was found to be involved in cell cycle pathways (Figure 2A). Examination of the expression of cell cycle genes across cancer types revealed that several kidney cancer samples had lower *CDK1* levels when compared to their matched normal parts (Figure 2B). However, *CDK1* upregulation alone was associated with poor overall patient survival in kidney renal clear cell carcinoma (KIRC) (log rank *p* = 4.13 × 10^−8^) and kidney renal papillary cell carcinoma (KIRP) (log rank *p* = 5.1 × 10^−15^).

In search of an alternative pathway that promotes tumor proliferation, we performed gene correlation studies in five kidney cancer patient samples with high *RNASEH2A* expression, low *CDK1* expression, and bad clinical outcomes (death). Twelve genes were found to be negatively correlated with *CDK1* expression, positively correlated with *RNASEH2A* expression, and associated with patient survival in KIRC or KIRP (Table S2). Further evaluation of the 12 gene expression levels in pooled TCGA RNAseq data of different cancer types revealed a strong negative correlation between *CD151* and *CDK1* expressions (Pearson *r* = −0.42, *p* < 0.0001, Figure 2C). Taken together, besides *RNASEH2A* upregulation, the *CD151*-related pathway might contribute to tumor growth in *CDK1*-low kidney cancers.

### 2.3. Analyses of the Interactions of RNASEH2A, CDK1, and CD151 Using Knockdown Studies in Four Kidney Cancer Cell Lines

**Figure 3:**
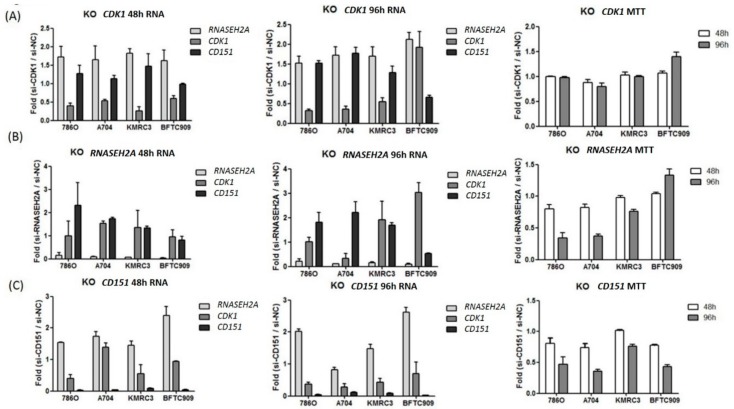
Kidney cancer cell line knockdown (KO) studies. (**A**) si-CDK1 (**B**) si-RNASEH2A (**C**) si-CD151. Gene expression fold changes at 48 h (left graphs) and at 96 h (middle graphs) post-transfection and knockdown effects on tumor proliferation measured by MTT assays (right graphs) are shown. Bars represent mean ± SD, *n* = 3.

**Figure 4:**
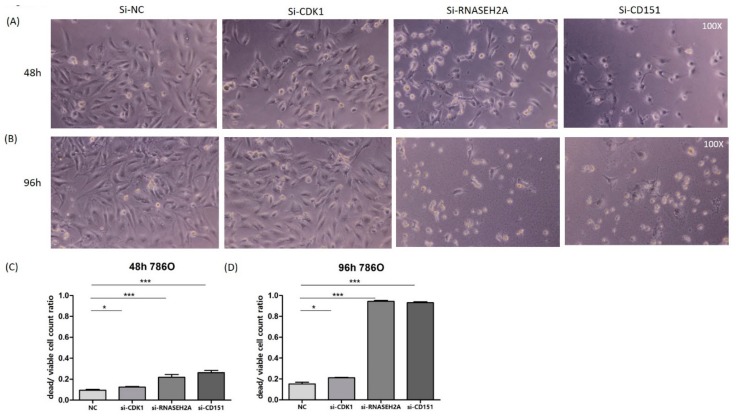
Cell viability assay in 786O cell line. (**A**,**B**) Light microscopy images of cells taken at 100× magnification, at 48 h (**A**) and 96 h (**B**) after transfection with si-NC (negative control), si-CDK1, si-RNASEH2A, or si-CD151. (**C**,**D**) Differential dead and viable cell count ratios in 786O cells transfected with si-NC, si-CDK1, si-RNASEH2A, or si-CD151 at 48 h (**C**) and 96 h (**D**). The bars represent mean ± SEM, *n* = 9; * *p* < 0.05; *** *p* < 0.0001 by Mann–Whitney *U* tests.

To study the interactions among *RNASEH2A*, *CDK1*, and *CD151* and their impact on tumor proliferation, we performed si-RNA knockdown studies on three ccRCC cell lines (786O, A704, KMRC3, all with *VHL* mutation) and one kidney urothelial carcinoma cell line (BFTC909, without *VHL* mutation). As shown in Figure 3A, *CDK1* knockdown resulted in the upregulation of *RNASEH2A* and *CD151* in all cell lines, however it did not significantly impair tumor proliferation (except for a mild effect on A704). Interestingly, in BFTC909, *CDK1* could not be knocked down after a 96-hour si-CDK1 transfection, and the tumor survival rate even increased. Furthermore, *RNASEH2A* knockdown resulted in *CD151* upregulation and decreased proliferation in all ccRCC cell lines. Elevated *CDK1* expression was observed in KMRC3 and BFTC909 cell lines 96 h after *RNASEH2A* knockdown; however, in contrast to KMRC3, *CD151* upregulation was not detected in BFTC909, and its cell proliferation was enhanced (Figure 3B). As for *CD151* knockdown experiments, the upregulation of *RNASEH2A* was observed in all kidney cancer cell lines and was associated with impaired tumor proliferation (Figure 3C). Cell viability assays were performed in ccRCC cell line 786O after transfection with si-CDK1, si-RNASEH2A, and si-CD151, which showed comparable results to the 3-(4,5-dimethylthiazol-2-yl)-2,5-diphenyltetrazolium bromide (MTT) assays (Figure 4).

### 2.4. Analyses of the Prognostic Values of CD151-Related Genes in RCC

**Figure 5:**
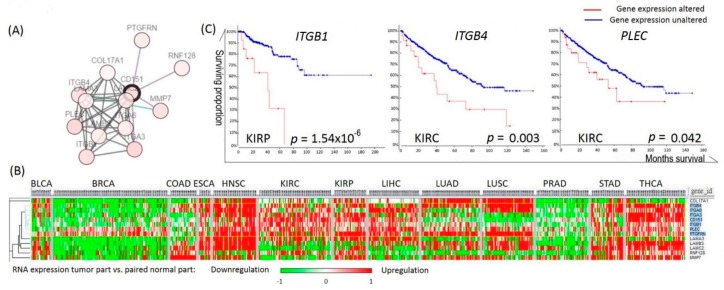
Genes correlated with *CD151* expression. (**A**) Interactome analysis of CD151 via cBioPortal. (**B**) Cluster analysis of genes correlated with *CD151* expression in different cancers. Genes that correlated the most with *CD151* are marked in blue. (**C**) Kaplan–Meier analyses of the overall survival in TCGA kidney cancer patients with or without altered gene expressions, calculated by cBioPortal.

Next, we performed *CD151* interactome analysis and cluster analysis to identify downstream genes in the *CD151*-related pathway (Figure 5A,B). The expression of *ITGB1*, *ITGB4*, and *PLEC* was found to be closely clustered with *CD151* expression, and their upregulation was associated with poor overall survival in KIRP or KIRC (Figure 5B,C).

### 2.5. Establishment of a Prognostication Scoring System for Survival Prediction in RCC

To establish a prognostication scoring system for predicting the ccRCC overall patient survival rate, Cox regression analyses using *RNASEH2A*, *CDK1*, and the mean of *ITGB1*, *ITGB4*, and *PLEC* expressions as variables were performed on TCGA KIRC total RNAseq data (tumor sample *n* = 533, with or without their normal counterparts). Cox regression coefficients (β) were used as weights for each gene expression score: Prediction system = (−0.65)* *RNASEH2A* expression z score + (−1.13) × *CDK1* expression z score + (−0.36) × *CD151* expression z score + (−0.40)* mean of (*ITGB1*, *ITGB4*, and *PLEC* expression z scores). The Cutoff Finder was used to determine the optimal cutoff score. 

**Figure 6:**
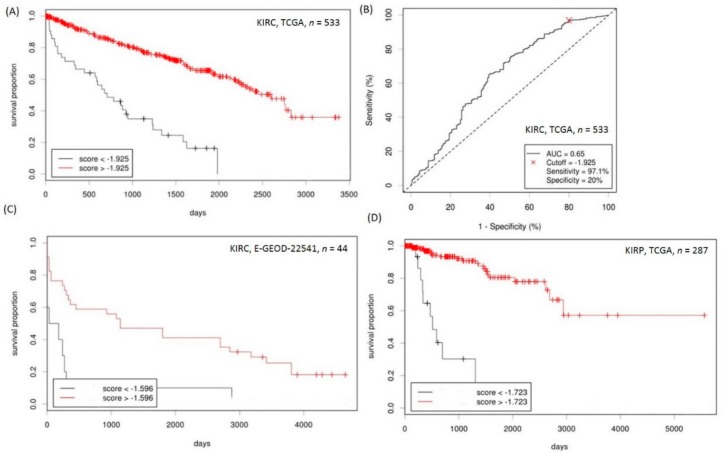
Prediction of kidney cancer overall patient survival rate using scores derived from cancer expression profiles of *RNASEH2A*, *CDK1*, *CD151*, *ITGB1*, *ITGB4*, and *PLEC*. (**A**) Kaplan–Meier analysis of the TCGA KIRC patients’ overall survival based on the optimal cutoff score derived from the cutoff finder. (**B**) Receiver operating characteristic (ROC) curve plotted on the optimal cutoff score of the TCGA kidney renal clear cell carcinoma (KIRC) cohort. (**C**) Kaplan–Meier analysis of disease-free survival of the E-GEOD-22541 clear cell renal cell carcinoma (ccRCC) validation cohort based on the optimal cutoff score. (**D**) Kaplan–Meier analysis of the TCGA kidney renal papillary cell carcinoma (KIRP) patients’ overall survival based on the optimal cutoff score.

In KIRC, patients with scores lower than the cutoff (−1.925) had a significantly worse overall survival rate compared to those with scores above the cutoff value (mean survival of 955.3 days versus 2242.2 days, hazard ratio (HR) = 4.53 (3.05–6.73), *p* = 2.2 × 10^−16^, Figure 6A). The receiver operating characteristic (ROC) curve that was plotted based on the optimal cutoff point is shown in Figure 6B. The prognostication score retained its independent predictive value when co-analyzed with clinical-pathological factors which showed a significant impact on the overall survival in univariate Cox regression analyses (adjusted HR for patients with lower prognostication score (< −1.925) = 2.68 (1.75–4.11), *p* < 0.0001, Table 1). In another ccRCC cohort, namely, the E-GEOD-22541 expression array data (*n* = 44), patients with scores lower than −1.596 also showed lower disease-free survival when compared to those with higher scores (mean survival of 390.0 days versus 1889.2 days, HR = 2.99 (1.07–8.35), *p* = 7.1 × 10^−4^, Figure 6C).

Furthermore, we applied the prediction system using the same coefficients as the TCGA KIRP (papillary RCC) cohort (*n* = 287) and we were able to clearly differentiate the patients with good or bad prognoses (mean survival for score < −1.723 versus score ≥ −1.723: 741.6 days versus 1623.7 days, HR = 13.19 (6.03–28.82), *p* = 4.4 × 10^−16^, Figure 6D). The prognostication score also retained its predictive value for the overall survival rate in papillary RCC (pRCC) patients after multivariate analyses considering other clinical-pathological parameters (adjusted HR for patients with score < −1.723 = 7.44 (3.01–18.41), *p* < 0.0001, Table 1).

## 3. Discussion

The above analyses highlight that, although *RNASEH2A* upregulation alone is not a universal cancer prognosis marker, it might form an alternative pathway with *CD151* to enhance tumor growth in *CDK1*-low renal cell carcinomas. A prognostication scoring system based on the expression levels of *RNASEH2A-*, *CDK1-*, and *CD151*-related genes could effectively predict the survival rate of RCC cancer patients (with a mean difference of four years in ccRCC).

*CDK1* promotes cell proliferation and survival and it is frequently overexpressed in cancer specimens [11]. Increased *CDK1* activity reportedly predicts RCC recurrence, and an association has been found between *CDK1* overexpression or hyperactivity and worse prognosis in ovarian, colorectal, and breast cancers [11,12,13,14]. However, the results from clinical trials for *CDK* inhibitors were mostly disappointing [15]. Similarly, in our study, *CDK1* knockdown in RCC cell lines did not have a pronounced effect on tumor growth. Interestingly, increased *RNASEH2A* and *CD151* expression was observed in si-CDK1-treated ccRCC cell lines and were was found in RCC patients with a low tumor *CDK1* level and bad clinical outcomes. This suggests that *RNASEH2A* and *CD151* have compensatory roles in promoting cell cycle progression and tumor growth. In contrast to *CDK1* knockdown, transfection of RCC cell lines with either si-RNASEH2A or si-CD151 resulted in impaired tumor proliferation. Upregulation of *CD151* and *RNASEH2A* was noted 48 h after si-RNASEH2A and si-CD151 knockdown, respectively, in all kidney cancer cell lines. Since synthetic dosage lethality (SDL) is defined by a combination of gene A overexpression and gene B underexpression and leads to cell death, our results suggest that *RNASEH2A* and *CD151* may have SDL interactions. Therefore, *CD151* is a potential drug target in *RNASEH2A* highly expressing tumors. Validation of the SDL relationships between *RNASEH2A* and *CD151* in other cancer types is needed.

*CD151* is known to form complexes with integrins and other transmembrane proteins and could be involved in cancer invasion and metastasis [16]. High *CD151* expression alone has been reported to predict cancer progression in ccRCC patients [17]. Our study identified that the expression of *CD151* downstream genes *ITGB1*, *ITGB4*, and *PLEC* may have additional prognostic values in kidney cancers. Consistent with this finding, simultaneous blocking of cdk1–cyclin B and integrin subtypes α5, α6, β4 using the drug sulforaphane was found to inhibit everolimus-resistant kidney cancer cell growth [18]. Furthermore, according to the potential SDL relationships between *CD151* and *RNASEH2A* identified by this study, *CD151*-high RCCs could be treated with *RNASEH2A* inhibitors.

RCC is among the 15 most common tumors around the world, and ccRCC is the most prevalent subtype [19]. The mortality rate of RCC is about 20–40%, partly due to metastasis at initial presentation, heterogeneity of cancer histology, as well as treatment resistance. Currently, the main prognostication system for metastatic RCC is the Memorial Sloan-Kettering Cancer Center (MSKCC) prognostic category, which is based on clinical and laboratory parameters [20]. Factors concerning tumor biology and the heterogeneous tumor genome and transcriptome are not included. Recently, mRNA and protein expression information was reported to have prognostic values in RCC [21,22]. Our study is the first to comprehensively analyze the genetic heterogeneity and potential SDL interactions that underlie the various clinical outcomes in *RNASEH2A*-high tumors. The derived prognostication scoring system that uses coefficients calculated from Cox regression analyses of the TCGA KIRC data is able to predict not only the prognosis of ccRCC, but also the survival rate of patients with the pRCC subtype. Our prediction system needs to be further validated in more kidney cancer cohorts.

## 4. Methods

### 4.1. Study Populations

The tumor-normal paired gene expression RNAseq data with more than 10 sample pairs were downloaded from The Cancer Genome Atlas (TCGA) data portal (https://tcga-data.nci.nih.gov/tcga/), including 14 cancer types (BLCA, BRCA, COAD, ESCA, HNSC, KICH, KIRC, KIRP, LIHC, LUAD, LUSC, PRAD, STAD, THCA). The full names and abbreviations of these 14 cancers are listed below: bladder urothelial carcinoma (BLCA), breast invasive carcinoma (BRCA), colon adenocarcinoma (COAD), esophageal carcinoma (ESCA), head and neck squamous cell carcinoma (HNSC), chromophobe kidney cancer (KICH), kidney renal clear cell carcinoma (KIRC), kidney renal papillary cell carcinoma (KIRP), liver hepatocellular carcinoma (LIHC), lung adenocarcinoma (LUAD), lung squamous cell carcinoma (LUSC), prostate adenocarcinoma (PRAD), stomach adenocarcinoma (STAD), and thyroid carcinoma (THCA). 

Furthermore, clinical data and all tumor RNAseq data collected from KIRC (ccRCC, *n* = 533) and KIRP (pRCC, *n* = 287) were also downloaded from TCGA. Moreover, microarray gene expression and clinical data of another clear cell renal cell carcinoma (ccRCC) cohort were downloaded from E-GEOD-22541 [23]. Comparisons of the clinical characteristics of the ccRCC cohorts are shown in the Table S1. This study has been approved on 23 June 2017, by China Medical University Hospital’s Institutional Research Ethics Committee (CMUH106-REC2-077).

### 4.2. Cell Line Studies

The kidney clear cell carcinoma cell lines 786O, A704, KMRC3, and kidney transitional cell carcinoma cell line BFTC909 were transfected with 10 nM siRNA for *RNASEH2A*, *CD151*, *CDK1*, or a negative control (purchased from MD Bio Inc., Taipei, Taiwan) using Lipofectamine RNAiMAX transfection reagent (Thermo Fisher Scientific-Invitrogen, Waltham, MA, USA), according to the manufacturer’s instructions. After 24 h, 48 h, 72 h, and 96 h of transfection, cell viability and proliferation were evaluated by MTT [3-(4,5-dimethylthiazol-2-yl)-2,5-diphenyltetrazolium bromide] assays. The reduced intracellular purple formazan crystals were dissolved by adding DMSO, and the absorbance was analyzed at 570 nm in a microtiter plate reader. The 786O cell line was also seeded on 24-well plates at 10^4^ cells/well, and cell culture images under a 100× light microscope were taken at 48 h and 96 h post-transfection. Cell viability was evaluated by counting the ratio of dead (rounded cells) versus viable cells (normal shape, attached to the plate).

### 4.3. RNA Isolation and Real-Time PCR Analyses

Total RNA was extracted by TRIzol Reagent (Thermo Fisher Scientific-Invitrogen, Waltham, MA, USA). Two microgram RNA was reverse-transcribed into cDNA using a High-Capacity cDNA Reverse Transcriptase Kit (Thermo Fisher Scientific-Applied Biosystems, Waltham, MA, USA) for real-time PCR analyses. The expression of *GAPDH* was used as an endogenous control. All primers were designed and synthesized by Genomics BioSci & Tech, Taipei, Taiwan. The following primer sequences were used: *RNASEH2A*-forward 5′-GAGAAAGAGGCGGAAGATGTTA-3′, *RNASEH2A*-reverse 5′-TCTTCCTGAGTCCCTCCTGA-3′; *CDK1*-forward 5′-TGGATCTGAAGAAATACTTGGATTCTA-3′, *CDK1*-reverse 5′-CAATCCCCTGTAGGATTTGG-3′; *CD151*-forward 5′-TCCTGCAGAGGAGTCGTTTC-3′, *CD151*-reverse 5′-CGGTGCCACATGTTGTCTT-3′.

### 4.4. Statistical Analysis

The RNA expression data were log_2_ transformed and normalized to z score (sample value minus data mean and then divided by data standard deviation). Kaplan–Meier analyses for the overall survival (OS) in patients with *RNASEH2A* overexpression were performed using the cBioPortal software (http://www.cbioportal.org/). Kaplan–Meier and Cox proportional hazards regression analyses for OS in patients with differential gene expression patterns were calculated via the MedCalc software version 14. The log-rank test *p* < 0.05 was used to determine the significance of the survival analysis.

For the establishment of the prognostication scoring system, the beta value of each gene upregulation (z score ≥ 0.5) was derived from the Cox regression analysis and was used as the weight to multiply the gene expression value (z score). The score was calculated on the ∑β × gene expression z score. Cutoff Finder version 2.1 [24] was used to determine the optimal cutoff scores and to plot the Kaplan–Meier survival curves, as well as the receiver operating characteristic (ROC) curves based on the cutoff values. Multivariate Cox regression analyses on prediction scores and significant clinical parameters (defined by univariate analyses) in RCC cohorts were performed via the SAS software v.9.3 (SAS Institute, Inc., Cary, NC, USA) to determine whether our score could predict RCC patient survival independently.

For categorical data, the Fisher’s exact test was used to evaluate the statistical significance. The Mann–Whitney U test was performed to calculate the differences in cell MTT proliferation assays and in cell viability assays. Gene expression correlation studies were analyzed by Pearson’s tests. Gene expression cluster analysis was performed via MORPHEUS software (https://software.broadinstitute.org/morpheus).

## 5. Conclusions

This study suggests that higher *RNASEH2A* and *CD151* gene expression might provide alternative pathways that enhance proliferation in *CDK1*-low tumors and presents a clinically applicable prognostication scoring system for RCC patients.

## Figures and Tables

**Table 1:** Univariate and multivariate Cox regression analyses to identify predictors of overall survival in ccRCC and papillary RCC (pRCC).

Variable	Group	Unadjusted HR (95% CI)	*p* Value	Adjusted HR (95% CI)	*p* Value
KIRC (*n* = 533)					
Age (per year)		1.02 (1.02–1.04)	<0.0001	-	-
Sex	Male	0.95 (0.69–1.31)	0.77	-	-
	Female	1			
Stage	High (stage III–IV)	4.28 (3.06–5.99)	<0.0001	-	-
	Low (stage I–II)	1			
Distant metastasis	M1	4.59 (3.32–6.33)	<0.0001	-	-
	M0	1			
Fuhrman grade	High (Gr 3–4)	2.85 (1.98–4.11)	<0.0001	-	-
	Low (Gr 1–2)	1			
Prediction score	Low (<−1.925)	4.53 (3.05–6.73)	<0.0001	2.68 (1.75–4.11)	<0.0001
	High (≥−1.925)	1			
KIRP (*n* = 287)					
Age (per year)		1.01 (0.98–1.04)	0.56	-	-
Sex	Male	0.72 (0.32–1.62)	0.43	-	-
	Female	1			
Stage	High (stage (III–IV)	4.86 (2.22–10.66)	<0.0001	-	-
	Low (stage I–II)	1			
Prediction score	Low (<−1.723)	13.19 (6.03–28.83)	<0.0001	7.44 (3.01–18.41)	<0.0001
	High (≥−1.723)	1			

HR: Hazard ratio.

## Supplementary Materials

Supplementary materials can be found at http://www.mdpi.com/1422-0067/19/6/1586/s1.
