# Supplementary material for: Prognostic Value of RNASEH2A-, CDK1-, and CD151-Related Pathway Gene Profiling for Kidney Cancers
Source: Int J Mol Sci. 2018 May 28;19(6):1586. doi: 10.3390/ijms19061586 (PMC6032436; doi:10.3390/ijms19061586)
Supplement: Supplementary file 1 [file ijms-19-01586-s001.pdf]

Table S1. Clinical characteristics of the ccRCC cohorts.

Variable	Group	TCGA-KIRC	E-GEOD-22541
Age	(years old)	61.1±12.2	60.1 ± 8.3*
Gender	Female	188	12
	Male	345	32
Fuhrman grade	G1	14	3
	G2	229	23
	G3	206	9
	G4	76	0
	Gx	3	0
	NA	5	9
Metastasis status	M0	422	24
	M1	79	20
	Mx	30	0
	NA	2	0

*The age information in E-GEOD-22541 was only available for 20 patients with M1 status.

Table S2. Correlation between gene expression levels and association with RCC patient overall survival (OS)

Gene	Correlation with CDK1 (*r*)	Correlation with RNASEH2A (*r*)	Associated with OS*
C7orf70	−0.72	0.31	Y
ANKRA2	−0.72	0.38	N
ENO2	−0.72	0.33	Y
GPX8	−0.68	0.42	Y
PSMB8	−0.68	0.41	N
DTX2	−0.66	0.39	Y
RFX2	−0.66	0.3	N
RAD9A	−0.63	0.33	Y
NRM	−0.63	0.39	Y
SAP30BP	−0.61	0.31	N
NUBP2	−0.61	0.33	N
TPI1	−0.6	0.33	N
MPG	−0.6	0.52	N
LPGAT1	−0.58	0.42	N
RPL38	−0.58	0.45	Y
C9orf89	−0.57	0.35	N
CD151	−0.57	0.3	Y
HLA-B	−0.54	0.34	N
C16orf42	−0.53	0.39	N

RUNX1	−0.52	0.52	Y
PSMB9	−0.52	0.52	Y
ZNF668	−0.51	0.52	Y
CTU2	−0.51	0.35	N
PRMT2	−0.5	0.43	N
PLXNA3	−0.5	0.37	Y

*Y: Patients with higher expression levels showed worse OS rate (log rank P ≦ 0.05 as calculated by cBioPortal).
